# The Role of Magnetic Resonance Imaging in a Difficult Case of Gallstone Ileus

**DOI:** 10.7759/cureus.33038

**Published:** 2022-12-28

**Authors:** Jonathan Tiong, Katherine Grant, Hamish Shilton

**Affiliations:** 1 General Surgery, Monash Health, Melbourne, AUS

**Keywords:** magnetic resonance imaging, cholecysto-duodenal fistula, small-bowel obstruction, gastrograffin, radiolucent object, gallstone ileus

## Abstract

Gallstone ileus is an uncommon condition that is difficult to diagnose clinically. Although several cases have been reported in the literature, radiolucent gallstones in the setting of gallstone ileus are an exceedingly rare occurrence, and we have not identified any authors who used magnetic resonance imaging (MRI) for the acute diagnosis of this condition. While an MRI is the gold standard for visualizing gallstones, inpatient MRIs are difficult to obtain, even in resource-rich settings. However, if given a high index of suspicion for gallstone ileus, it is pertinent to advocate for an inpatient MRI despite a resolution of patients’ symptoms due to the nature of the disease symptomology.

## Introduction

Gallstone ileus is a rare condition caused by the impaction of the gallstone within the intestinal tract and constitutes 0.095% of small bowel obstructions [1]. The pathogenesis is explained by the formation of a bilioenteric fistula, which begins as a result of an impacting calculous within the gallbladder wall against the enteric lumen causing vascular insufficiency. Common risk factors are female sex (70%), age >65 years, and a history of multiple comorbidities [2].

Due to the nature of the obstruction, it is frequently described as a “tumbling” advancement, an alternating aggravation and resolution of the ileus as the calculous moves along the intestinal lumen. Patients describe intermittent abdominal pain, bloating, and distention. However, obstructive symptoms, including vomiting, can predominate in the emergency setting [3].

Computed tomography scans are a reliable tool and often the first imaging modality in these patients. However, in certain cases, they may not visualize the gallstone. Magnetic resonance imaging, on the other hand, is the gold standard imaging modality for radiolucent gallstones; however, in Asia-Pacific, it is infrequently used as the first-line investigation, especially in intra-abdominal surgical conditions due to scarcity and cost [3].

Surgical management remains the mainstay of treatment, with three predominating procedures: enterolithotomy, concurrent enterolithotomy, cholecystectomy and fistula repair (in a single operation), or a two-stage procedure comprising enterolithotomy first and cholecystectomy later [2].

This article was previously presented as a meeting abstract at the 13th International Conference on Clinical Gastroenterology & Hepatology Meeting on March 17, 2022.

## Case presentation

A 69-year-old male presented with a one-day history of epigastric pain associated with vomiting. He had no history of biliary disease or medical comorbidities and did not give a history consistent with biliary colic. Bloodwork revealed an inflammatory rise (white cell of 15.5 x 109, C-reactive protein of 10 mg/L), a raised bilirubin of 38 mcmol/L, and normal liver function tests (LFT).

**Figure 1:**
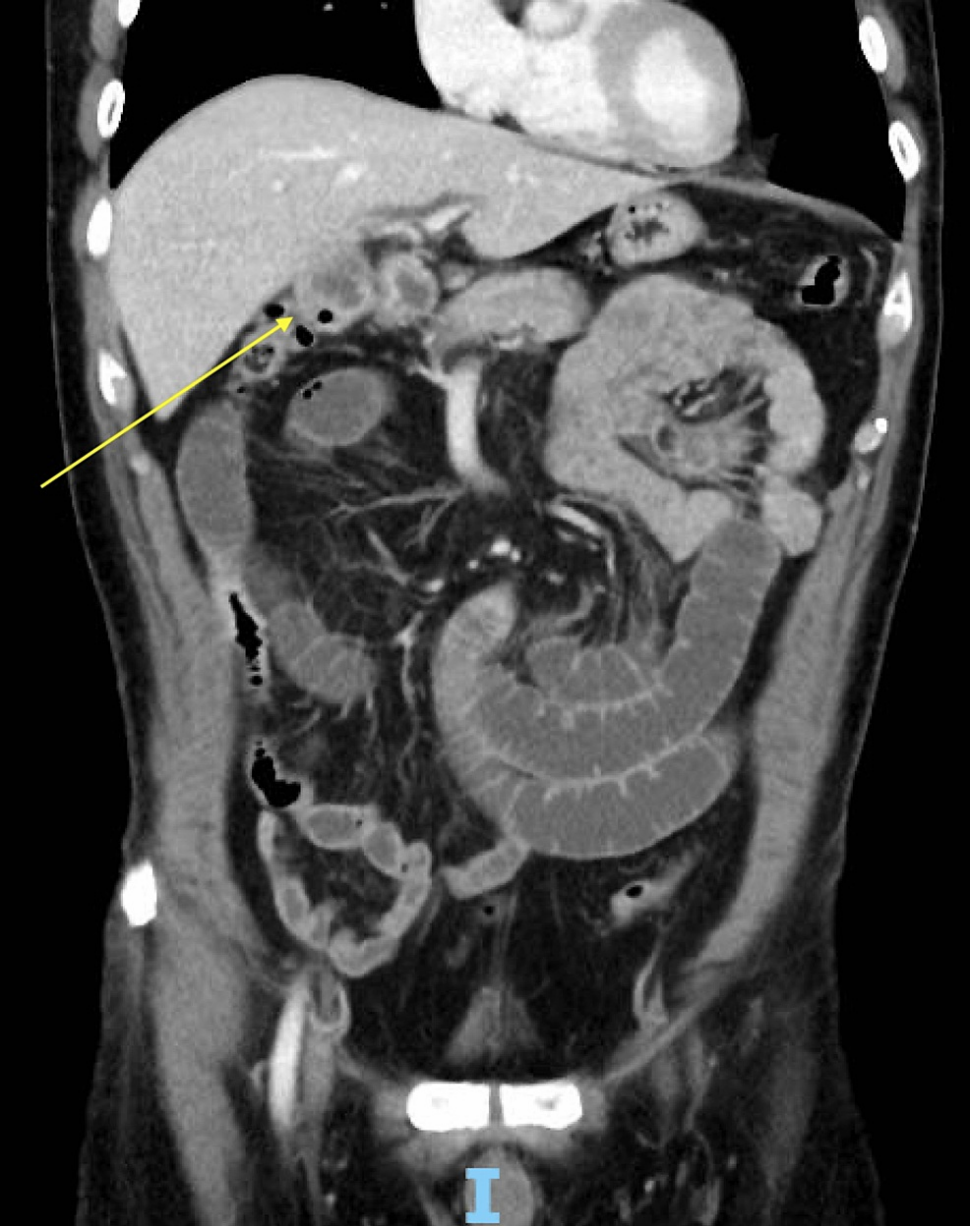
The site of the cholecystoduodenal fistula A small bowel obstruction with no clear transition point was noted.

Initial computed tomography (CT) scan of the abdomen reported findings suspicious for chronic cholecystitis and secondary cholecystoduodenal fistula, with mild dilatation of the common bile duct. CT also revealed prominent dilated loops of the small bowel with air-fluid levels and distal transition points concerning for small bowel obstruction (Figure 1).

**Figure 2:**
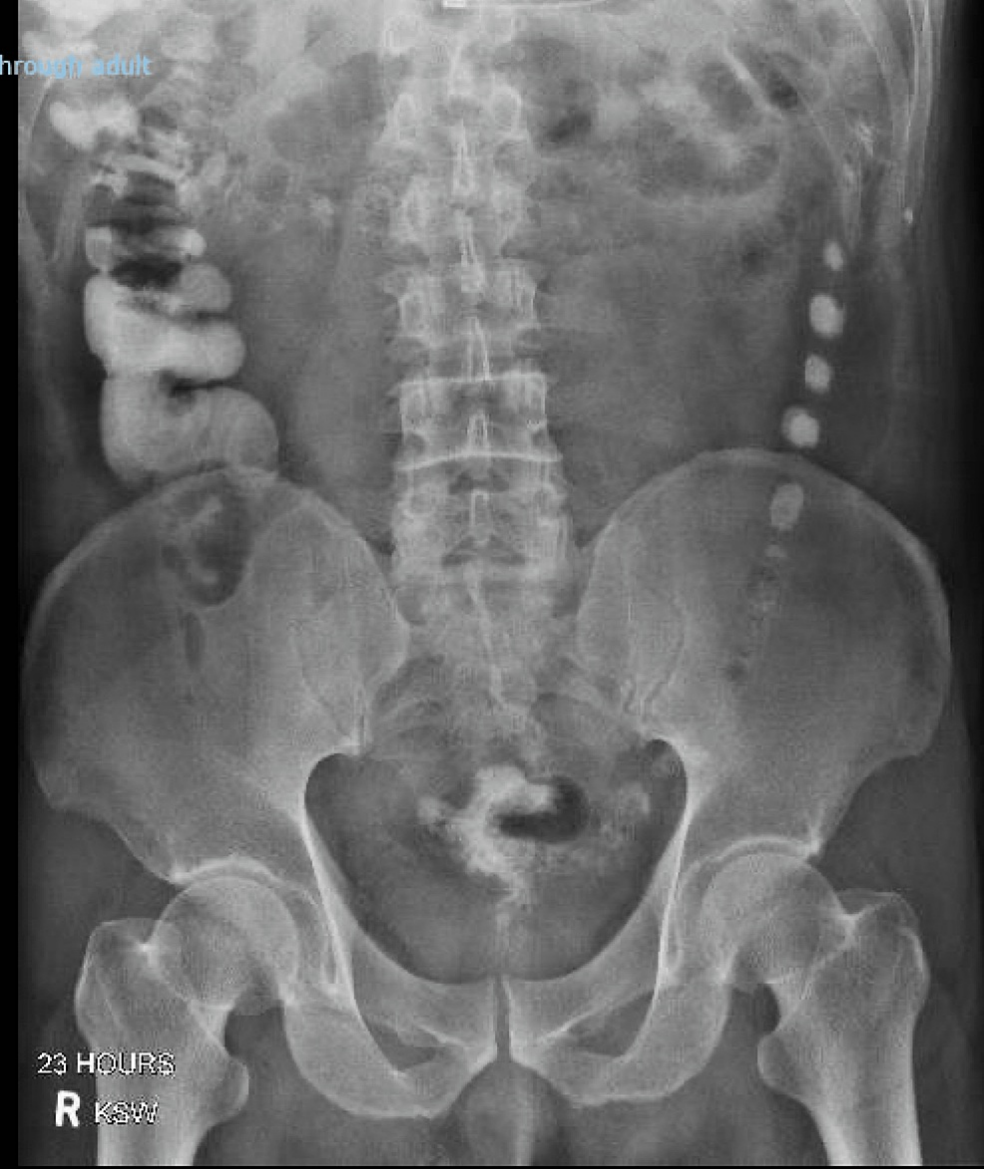
Gastrograffin follow-through (GGFT) showing resolving bowel obstruction with free flow of contrast into the large bowel 23 hours post gastrograffin

He was initially treated with antibiotics, a nasogastric tube, and diet restriction with a plan for magnetic resonance enterography (MRE). Given delays in the MRE for logistical reasons, a gastrograffin follow-through (GGFT) study was performed the next day, showing a resolving bowel obstruction, with contrast flowing into the colon (Figure 2). Bilirubin had also normalized to 18 mcmol/L. The patient opened his bowels, had no further pain, and thus was discharged with a plan for an outpatient MRE and follow-up with our hepatobiliary team in six weeks.

He re-presented one day later with worsening symptoms of the right upper quadrant and epigastric pain and vomiting. Examination revealed he was Murphy’s negative but tender with a mildly distended abdomen. LFTs were again normal, with a slightly raised bilirubin of 24 mcmol/L. He was kept fasting and given antibiotics.

**Figure 3:**
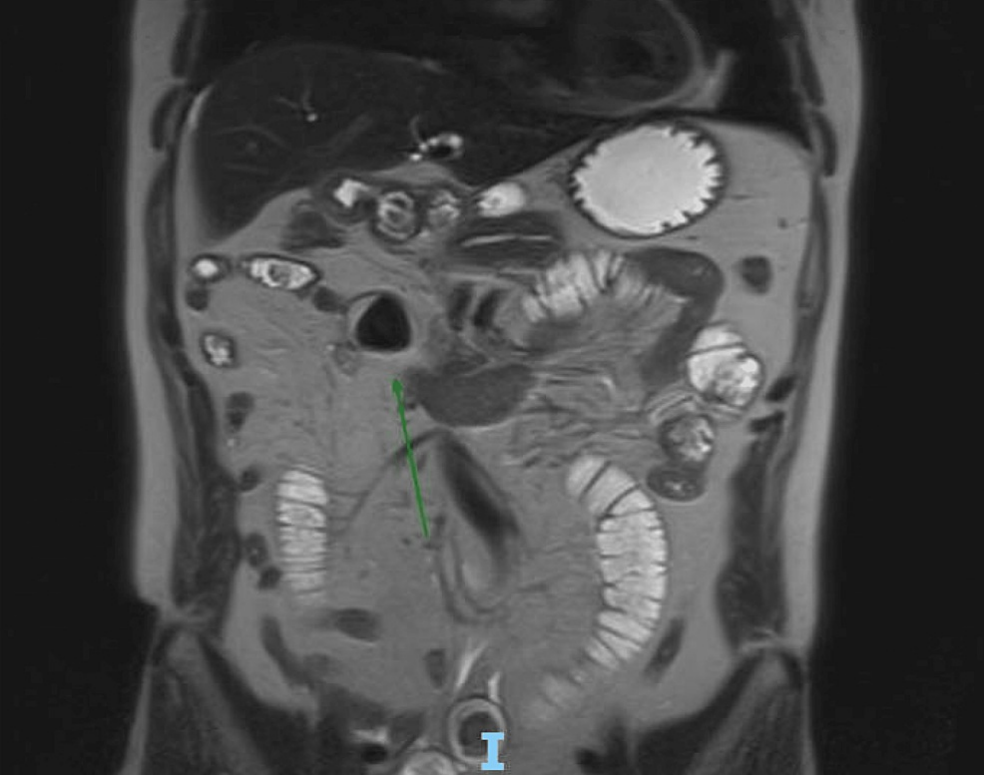
Magnetic resonance enterography (MRE) showing biliary calculi in the area of the mid ileum measuring 24.5 mm

An urgent MRE was performed showing gallstone ileus with a 25 mm calculus in the mid-small bowel and bowel obstruction (Figure 3). It also demonstrated cholelithiasis, choledocholithiasis, and a cholecystoduodenal fistula.

**Figure 4:**
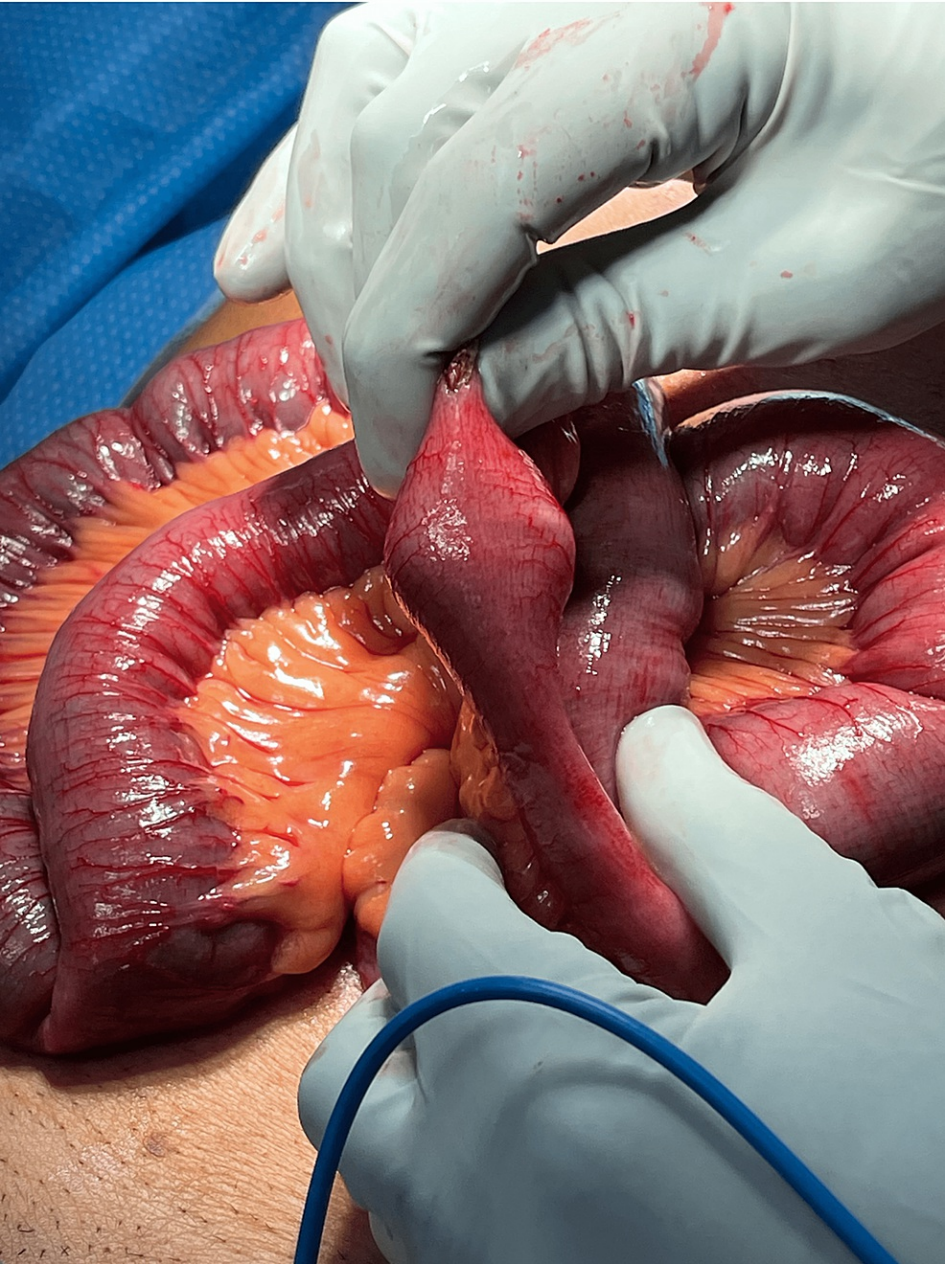
Intraoperative findings Note the dilated proximal loops of the bowel.

The patient subsequently underwent an exploratory mini-laparotomy revealing a dilated proximal small bowel dilatation with collapsed distal loops. Inspection of the gallbladder and liver demonstrated dense omental adhesions. An obstruction was found in the mid ileum and an enterotomy was created at the site revealing a 25 mm gallstone (Figure 4). After the removal of the calculus, the small bowel was milked in its entirety. The lumen was closed transversely and a leak test confirmed adequate closure.

The patient developed an ileus postoperatively, with a follow-up CT abdomen on Day 6 postoperatively revealing no bowel obstruction. After tolerating a full ward diet and reporting active bowel movements, he was managed conservatively and discharged on Day 8.

On his one- and eight-month postoperative follow-up, the patient reported no new or ongoing symptoms of abdominal discomfort and tolerated a full ward diet. The patient was offered a definitive cholecystectomy and fistula repair. However, he decided not to proceed and opted to monitor and re-present to his local emergency department if similar symptoms develop.

## Discussion

Gallstone ileus is an uncommon diagnosis and rare complication of cholelithiasis constituting less than 0.1% of small bowel obstructions [1]. Vascular insufficiency is posited to play a role in the formation of a bilioenteric or bilio-colonic fistula by way of the impacting calculus. Although risk factors for gallstone ileus are poorly defined, patients are often female, comorbid, and overweight [2]. Symptoms often vary due to the movement of the stone through the intestinal tract. Patients often present with intermittent abdominal pain, distention, vomiting, and fevers. Symptoms earlier in the history may mimic biliary colic while presenting complaints often resemble bowel obstructions [3]. Physical examination often reveals generalized abdominal pain and distention.

Because of the typically vague and wide range of symptoms, imaging can be broadly prescribed. Abdominal radiographs may show pneumobilia, small bowel obstruction, and an aberrantly located gallstone, otherwise known as Rigler’s triad [4]. The widespread use of CT in this patient population has yielded a sensitivity and specificity of 93% and 100%, respectively, and diagnostic accuracy of 99%. The presence of small bowel obstruction is well-defined. However, gallstones are not often seen due to the nature of their composition. Gallstone calculi are largely composed of cholesterol and bile salts, which are typically radiolucent. However, transition points can be well defined at the terminus of the stone [5].

While our patient displayed symptoms of bowel obstruction, blood tests and imaging were not definitive. Despite the CT suggesting a fistula, no stone was found within the small bowel and a transition point was not well-defined. Rigler’s triad was also not demonstrated on abdominal radiographs. Furthermore, his symptoms completely resolved with the administration of a GGFT, which again did not illustrate pneumobilia or an obstructing stone. Complete resolution is uncommon, and typically involves evacuation of the culprit calculus, which was not demonstrated in this case.

Magnetic resonance imaging (MRI) is well-regarded as the gold standard for detecting radiolucent and isoattenuating stones otherwise missed on X-ray and CT scans [6]. However, does not often play a role in the acute setting for intra-abdominal surgical presentations, especially in the Asia Pacific. And though gallstone ileus has been described in the literature, there have been no reports of MRE being used in the acute setting for diagnostic purposes [7]. Thus, if given a high index of suspicion for gallstone ileus, gallstone ileus warrants an expeditious MRE to confirm the diagnosis due to the urgency of management, as highlighted in our patient.

The management of gallstone ileus is contentious. There are three main operative strategies. Enterolithotomy, concurrent enterolithotomy, cholecystectomy, and fistula repair, or a two-stage procedure to the aforementioned [2]. Our patient received an enterolithotomy alone, which is well-known to be a safe and effective strategy for managing acute episodes [8]. However, it carries a 5% risk of recurrence given the presence of an existing fistula [9]. Concurrent procedures are more invasive and confer higher mortality and morbidity [8]. A two-stage procedure has been posited for younger patients with fewer/no comorbidities to prevent their risk of recurrence [10].

## Conclusions

This case was a demonstration of a difficult diagnosis of a radiolucent gallstone ileus with a series of negative CT and radiograph findings with a rapidly resolving bowel obstruction after GGFT. Clinicians with a high index of suspicion should advocate for inpatient MRE to confirm the diagnosis and be cognisant that the resolution of symptoms may not suggest the resolution of pathology. Enterolithotomy is safe and highly successful. Follow-up for consideration of definitive management is warranted given the risk of recurrence.
